# Evaluation of Plan Robustness Using Hybrid Intensity-Modulated Radiotherapy (IMRT) and Volumetric Arc Modulation Radiotherapy (VMAT) for Left-Sided Breast Cancer

**DOI:** 10.3390/bioengineering9040131

**Published:** 2022-03-24

**Authors:** Zhen Ding, Qi Zeng, Kailian Kang, Meiling Xu, Xiaoyong Xiang, Chenbin Liu

**Affiliations:** Department of Radiation Oncology, National Cancer Center/National Clinical Research Center for Cancer/Cancer Hospital & Shenzhen Hospital, Chinese Academy of Medical Sciences and Peking Union Medical College, No. 113 Baohe Road, Longgang District, Shenzhen 518116, China; dingzhen0909@163.com (Z.D.); zengqi1203@163.com (Q.Z.); kangkailian@163.com (K.K.); xmlmath@163.com (M.X.)

**Keywords:** left-sided breast, robustness, volumetric modulated radiation therapy, hybrid intensity-modulated radiotherapy, tumor control probability, normal tissues complication probability

## Abstract

Purpose: We aim to evaluate the robustness of multi-field IMRT and VMAT plans to target motion for left-sided BC radiotherapy. Methods: The 7-field hybrid IMRT (7F-H-IMRT) and 2-arc VMAT (2A-VMAT) plans were generated for ten left-sided BC patients. Shifts of 3 mm, 5 mm, and 10 mm in six directions were introduced and the perturbed dose distributions were recalculated. The dose differences (∆D) of the original plan and perturbed plan corresponded to the plan robustness for the structure. Results: Higher ∆D_98%_, ∆D_95%_, and ∆D_mean_ of CTV were observed in 2A-VMAT plans, which induced higher tumor control probability reductions. A higher ∆Dmean of CTV Boost was found in 7F-H-IMRT plans despite lower ∆D_98%_ and ∆D_95%_. Shifts in the S-I direction exerted the largest effect on CTV and CTV Boost. Regarding OARs, shifts in R, P, and I directions contributed to increasing the received dose. The 2A-VMAT plans performed better dose sparing, but had a higher robustness in a high-dose volume of the left lung and heart. The 2A-VMAT plans decreased the max dose of LAD but exhibited lower robustness. Conclusion: The 2A-VMAT plans showed higher sensitivity to position deviation. Shifts in the S-I direction exerted the largest effect for CTV and CTV Boost.

## 1. Introduction

Hypofractionated whole-breast radiotherapy (WBRT) following breast-conserving surgery has been widely used for equivalent local control, survival, and toxicity to conventional fractionation in published articles [1,2,3,4]. The simultaneous integrated boost (SIB) technique has shown advantages by using different dose levels to treat the tumor bed in a single treatment session, rather than sequential boost delivery [5,6]. Cardioprotective strategies to further mitigate the injurious effects of radiotherapy (RT) are paramount as cardiac complications significantly affect the overall survival of breast cancer (BC) patients [7]. The study by Afifi et al. [8] showed that BC remained the main cause of death, and other non-cancer causes of death (mainly heart and cerebrovascular diseases) represented a significant number of deaths, among patients with BC. The risk of a major coronary event increased linearly with the mean dose to the heart [9,10] due to the deep location of the target area. The risk depends on the local radiation dose, which indicated the potential of reducing the risk by optimizing the dose distribution in the heart [11].

Hybrid intensity-modulated radiotherapy (IMRT) [12], which added 3–5 IMRT fields based on two tangential fields, obtained an improved dose distribution and organ-at-risk (OAR) sparing compared to the traditional tangential field-in-field technique. Highly optimized volumetric arc modulation radiotherapy (VMAT) further improved dose distribution, heart and LAD sparing, though with an unexpected large low dose volume [13,14]. With increased plan complexity, the risks of inaccurate dose calculations and treatment delivery are elevated [15] compared to non-modulated plans. More complex plans require smaller and more irregular beam apertures, larger tongue-and-groove effects, and small sizes but with greater amounts of sub-fields by modulating the position of the multi leaf collimator [15]. Tumor position deviations caused by variations in patient geometry and respiratory motion may exert enlarged effects [16,17]. Plan robustness refers to the capability to deal with all the uncertainties, such as setup errors, patient anatomical change, etc. 

To the best of our knowledge, there are few studies about the evaluation of physical dose robustness and biological dose distributions using photon radiotherapy [18]. As radiotherapy treatment plans have increased in complexity, the need for plan robustness quantification has increased markedly. In this study, we aimed to assess the plan robustness of hybrid IMRT and VMAT techniques for BC treatments. Dose-volume histogram (DVH) band width was used to quantify the dose delivery inaccuracy caused by tumor motion. To evaluate the biological dose differences, tumor control probability (TCP) and normal tissues complication probability (NTCP) models were applied. 

## 2. Methods

### 2.1. Ethics Approval and Consent to Participate

The study was approved by the institutional review board of the National Cancer Center/National Clinical Research Center for Cancer/Cancer Hospital & Shenzhen Hospital (Approval Code: 2020-23; Approval date: 23 March 2020).

### 2.2. Patient Selection and Delineation

Ten patients who underwent adjuvant radiotherapy after left-sided breast-conserving surgery were selected. The patient characteristics are listed in Table 1. All patients were immobilized using a breast bracket in a supine position (CIVCO Medical Solutions, Orange City, IA, USA). CT images with a slice thickness of 5.0 mm were acquired using a 16-slice CT scanner (GE Discovery RT, GE Healthcare, Chicago, IL, USA). The clinical target volume (CTV), clinical target volume boost (CTV Boost), and OARs were delineated by an experienced oncologist and approved by a senior physician. The PTV and PTV Boost was generated by applying 5 mm radial and longitudinal margins to the CTV and CTV Boost, respectively. 

### 2.3. The 7-Field Hybrid IMRT and VMAT Plans 

The 7-field hybrid IMRT (7F-H-IMRT) and 2-arc VMAT (2A-VMAT) plans were generated using a Varian Eclipse (13.6 Version) treatment planning system (TPS), modeled with the VitalBeam (Varian, Palo Alto, CA, USA) linear accelerator (LINAC). The prescription doses were 43.5 Gy for PTV and 49.5 Gy for PTV Boost. The 7F-H-IMRT plan consisted of 2 tangential conformal fields and 5 IMRT fields with fixed gantry angles of 320°, 340°, 30°, 80°, and 110°. The prescription dose in the 7F-H-IMRT plan was 34.8 Gy for tangential fields and 14.7 Gy for IMRT fields. For the 2A-VMAT plan, two partial arcs were used and the gantry angle ranged from 300° to 160°. Collimator angles were specified at ±5°. The same optimization objective, convolution optimization, and iterative optimization were used. 

### 2.4. Dosimetric Evaluation

For PTV and PTV Boost, the 95% dose coverage (D_95%_), 98% dose coverage (D_98%_), 2 cm^3^ dose coverage (D_2cc_), and PTV Boost mean dose (D_mean_) were evaluated. For OARs, V_40__Gy_, V_20__Gy_, V_5__Gy_, D_mean_ of ipsilateral lung (Lung L) and heart; V_5__Gy_, D_mean_ of contralateral lung (Lung R) and the contralateral breast (Breast R), and D_max_ and D_mean_ of LAD were evaluated. D_x%_ represented the dose (in Gy) received by x% of the volume, V_yGy_ was the volume (in percentage) received by y Gy, D_2cc_ was the dose (in Gy) received by a volume of 2 cm^3^. D_max_ and D_mean_ represented the max and mean dose.

### 2.5. TCP and NTCP

We used the tumor control probability (*TCP*) and normal tissue complication probability (*NTCP*) models to evaluate the biological effects. The Schultheiss logit model proposed by Niemierko [19] was adopted to calculate the *TCP* according to Equation (1) with the parameters of *TCD*_50_= 30.89 Gy, *γ*_50_ = 1.3 [20].

(1) $$TCP=\frac{1}{1+{\left(\frac{{TCD}_{50}}{EUD}\right)}^{4\gamma_{50}}}$$

*TCD*_50_ is the radiation dose that locally controls 50% of the tumor cells when the dose is homogeneously irradiated. *γ*_50_ describes the slope of dose–response curve at the value of *TCD*_50_. We calculated *NTCP* [20] according to Equation (2):

(2) $$NTCP=\frac{1}{\sigma \sqrt{2\pi }}\overset{EUD}{\underset{-\infty }{\int }}e^{-\left(\frac{{\left(x-TD_{50}\right)}^{2}}{2\sigma^{2}}\right)}dx$$

*σ* was calculated by Equation (3):

(3) $$\sigma =mTD_{50}$$

The equivalent uniform dose (*EUD*) was calculated according to Equations (4) and (5):

(4) $$EUD={\left(\underset{i=1}{\sum }v_{i}EQD_{2i}^{\frac{1}{n}}\right)}^{n}$$

(5) $$EQD_{2i}=d_{i}\frac{\left(\frac{\alpha }{\beta }+\frac{d_{i}}{n_{f}}\right)}{\left(\frac{\alpha }{\beta }+2\right)}$$

*EQD*_2_ represented the equivalent dose in 2 Gy per fraction, which depended on the fraction size and *α/β* ratio for each case. *TD*_50_ is the tolerance dose yielding a 50% complication rate in the normal organ. v_i_ is the volume at dose *D_i_*. Parameter m and n are specific dose–response constants [21]. *n_f_* is the number of fractions. The *α/β* values of breast and lung are 4.0, and 3.7 for heart [21]. 

### 2.6. A Robustness Quantification Method

All original plans were normalized so that ≥95% of the PTV Boost received 100% of the prescription dose. To simulate the dose variations due to tumor shift, eighteen perturbed dose distributions were calculated for the 2A-VMAT and 7F-H-IMRT plans, respectively, by shifting the isocenter from its reference point in the superior–inferior (S-I), left–right (L-R), and anterior–posterior (A-P) directions by ±3, ±5, and ±10 mm. 

We adopted the DVH band width as the robustness quantification method in this study. The dosimetric parameters in the treatment and perturbed plans are shown in the dose–volume histogram (DVH) curves. The differences in the dosimetric parameters (∆D_x%_ and ∆V_y Gy_) between the perturbed scenario and nominal scenario were calculated. The absolute value of the difference corresponded to the plan robustness for the structure. A smaller value indicates better robustness for certain dosimetric parameters. We also calculated the TCP and NTCP reduction (∆TCP and ∆NTCP).

### 2.7. Statistical Analysis

The Wilcoxon signed-rank test was performed using IBM SPSS v.20 software (IBM Incorporate, Armonk, NY, USA). A *p* value of less than 0.05 (* *p* < 0.05) was considered statistically significant. 

## 3. Results

### 3.1. Dosimetric Parameters

Plan Evaluation: The dosimetric parameters of PTV, PTV Boost, CTV and CTV Boost are shown in Table 2. Both the 2A-VMAT and 7F-H-IMRT plans were clinically acceptable with adequate target coverage. No significant differences were observed in D_2cc_ (*p* = 0.06), D_98%_ (*p* = 0.19) and D_mean_ (*p* = 0.11) of PTV Boost. For CTV Boost, the 2A-VMAT plans performed higher D_95%_ (*p* = 0.69), D_mean_ (*p* = 0.13) and TCP (*p* = 0.11). PTV performed higher D_mean_ (*p* = 0.23) in 2A-VMAT plans with a higher D_98%_ (*p* = 0.43) and a lower D_95%_ (*p* = 0.19) than those in 7F-H-IMRT plan. As to CTV, significantly higher D_98%_ (** *p* = 0.002), D_95%_ (** *p* = 0.002) and D_mean_ (* *p* = 0.05) were seen in 2A-VMAT plans. CTV exhibited a higher TCP (*p* = 0.13) in 2A-VMAT plans. 

Table 3 showed the dosimetric parameters of all the OARs. The 2A-VMAT plans exerted significantly better protection of Lung L, with a lower V_40__Gy_ (** *p* = 0.002), V_20Gy_ (** *p* = 0.001), and D_mean_ (* *p* = 0.04), along with a higher V_5 Gy_ (* *p* = 0.05). A decreased NTCP of Lung L indicated the improved dose sparing by VMAT. The V_40__Gy_ (** *p* = 0.002), V_20__Gy_ (** *p* = 0.001) and D_mean_ (** *p* = 0.001) of Heart in the 2A-VMAT plans were significantly lower. As a consequence, a significantly lower NTCP (** *p* = 0.002) was obtained. D_max_ (** *p* = 0.002) and D_mean_ of LAD (** *p* = 0.002) were significantly reduced. V_5__Gy_ and D_mean_ of Lung R and breast R in the 2A-VMAT plans were significantly higher than 7F-H-IMRT plans, due to the larger volume of low dose coverage. Higher NTCP (** *p* = 0.002) of lung R were observed in the 2A-VMAT plans.

### 3.2. Plan Robustness Evaluation

**Figure 1:**
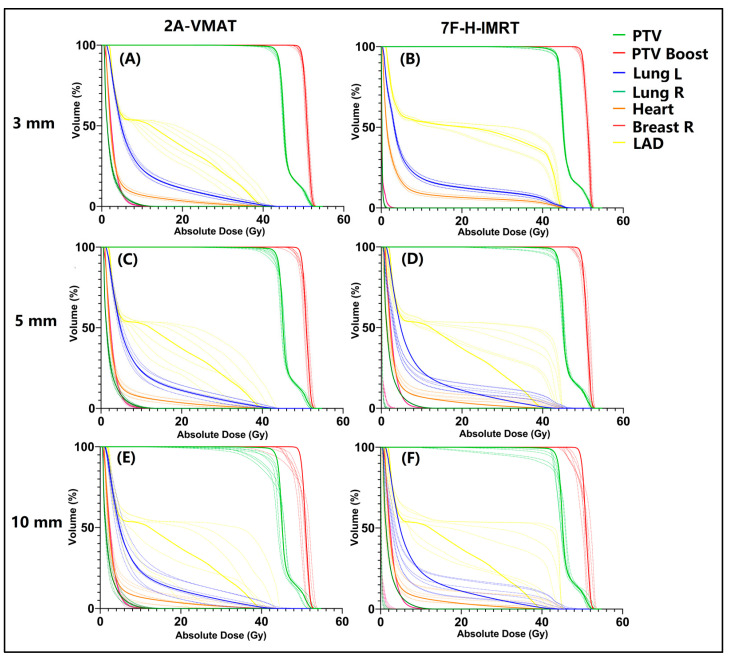
A sample of dose–volume histograms (DVHs) with different position shifts in 2A-VMAT and 7F-H-IMRT plans. (**A**) 3 mm shift in 2A-VMAT plan; (**B**) 3 mm shift in 7F-H-IMRT plan; (**C**) 5 mm shift in 2A-VMAT plan; (**D**) 5 mm shift in 7F-H-IMRT plan; (**E**) 10 mm shift in 2A-VMAT plan; (**F**) 10 mm shift in 7F-H-IMRT plan. PTV, planning target volume; PTV Boost, planning target volume boost; CTV, clinical target volume; CTV Boost, clinical target volume boost; Lung L, left lung; Lung R, right lung; Breast R, right breast. 2A-VMAT, 2-arc volumetric arc modulation radiotherapy; 7F-H-IMRT, 7-field hybrid intensity-modulated radiotherapy.

When the position shifts in the six directions were introduced, six perturbed plans were obtained for each shift. A sample of the dose–volume histograms (DVHs) is shown in Figure 1. In a 3 mm shift, both the 2A-VMAT and 7F-H-IMRT plans showed superior robustness. The larger the value of the position shift, the less robust the plans were observed in both two techniques. We applied a plan robustness quantification method, in which dose difference ∆D was calculated and corresponded to the plan robustness for the structure. 

The ∆D_98%,_ ∆D_95%_ and ∆D_mean_ of CTV are shown in Table 4. For a 3 mm shift, mean ∆D_95%_ and ∆D_mean_ were less than 1.0 Gy in both 2A-VMAT and 7F-H-IMRT plans in all directions. The 7F-H-IMRT plans achieved lower mean ∆D_95%_ and ∆D_mean_ than those in 2A-VMAT in most of the directions, except for the A and P directions. The mean ∆D_98%_ of the 2A-VMAT plans in S direction was 1.57 Gy, obviously higher than 7F-H-IMRT plans. For a 5 mm shift, the dose differences were enlarged, especially in ∆D_98%_ and ∆D_95%_ of 2A-VMAT plans. The ∆D_98%_ of 2A-VMAT plans reached 3.37 Gy and 2.77 Gy in the S and I directions, respectively, over 10 times higher than those in the 7F-H-IMRT plans (0.21 Gy and 0.17 Gy). The 5 mm shift in the R and I directions induced ∆D_95%_ of 1.22 Gy and 2.45 Gy in the 2A-VMAT plans. The ∆D_98%,_ ∆D_95%_ and ∆D_mean_ of CTV in 7F-H-IMRT plans were all less than 1.0 Gy. Further enlarged dose differences were seen in the 10 mm shift perturbation. The largest dose differences were observed for ∆D_98%_ of the 2A-VMAT plans. The ∆D_98%_ in S-I directions exceeded 10.0 Gy. No marked ∆D_98%_ were seen except in the S direction. The mean ∆D_95%_ in the R, S, and I directions were 5.70 Gy, 1.89 Gy, and 9.14 Gy, however, values no larger than 1.0 Gy were observed in the 7F-H-IMRT plans. Higher ∆D_mean_ of CTV in were observed in 2A-VMAT plans in all directions. The results indicated that the 7F-H-IMRT plans showed a stronger robustness than the 2A-VMAT plans. The shifts in the S-I directions exerted the largest effects. 

The ∆D_98%_, ∆D_95%_ and ∆D_mean_ of CTV Boost are shown in Table 5. For a 3 mm perturbation for 2A-VMAT plans, less than 1.0 Gy of mean ∆D_98%,_ ∆D_95%_ and ∆D_mean_ of CTV Boost were observed, except for ∆D_98%_ in the S direction. The mean ∆D_95%_ and ∆D_mean_ in the 7F-H-IMRT plans were lower than those in 2A-VMAT in most of the directions, except for the A and P directions. For a 5 mm shift, amplified dose differences were seen in ∆D_98%_ of CTV Boost in 2A-VMAT plans, while the 7F-H-IMRT plans showed a higher robustness. As to ∆D_95%_ and ∆D_mean_, a shift in L–R and S–I directions induced greater dose differences. It was noticeable that the 7F-H-IMRT plans in all directions showed a higher ∆D_mean_. The ∆D_mean_ of 7F-H-IMRT plans was 2.65 Gy, almost three times greater than that in the 2A-VMAT plans. A 10 mm shift further enlarged the dose differences. The ∆D_98%_ of CTV Boost in all directions in 2A-VMAT was obviously greater than those in the 7F-H-IMRT plans. A 10 mm shift in the A direction caused a higher ∆D_95%_ in the 7F-H-IMRT plans. The 7F-H-IMRT plans in all directions showed higher values of ∆D_mean_, even though A-VMAT plans exerted a greater ∆D_98%_ and ∆D_95%_. Similarly, the shifts in the S-I direction exerted greatest effect for CTV Boost.

**Figure 2:**
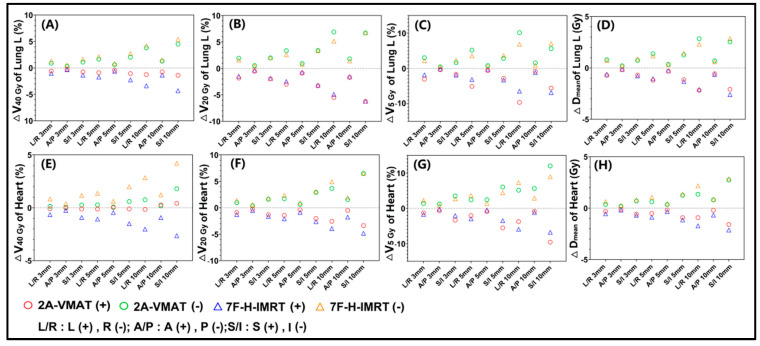
Dose difference between the reference and perturbed 2A-VMAT and 7F-H-IMRT plans for different isocenter shifts. (**A**) ∆V_40__Gy_ of Lung L; (**B**) ∆V_20__Gy_ of Lung L; (**C**) ∆V_5 Gy_ of Lung L; (**D**) ∆D_mean_ of Heart; (**E**) ∆V_40__Gy_ of Heart; (**F**) ∆V_20__Gy_ of Heart; (**G**) ∆V_5 Gy_ of Heart; (**H**) ∆D_mean_ of Heart. Lung L, left lung. V_y Gy_, the volume (in percentage) received by y Gy; D_mean_, the mean dose; D_max_, the max dose; 2A-VMAT, 2-arc volumetric arc modulation radiotherapy; 7F-H-IMRT, 7-field hybrid intensity-modulated radiotherapy; L, left; R, right; A, anterior; P, posterior; S, superior; I, inferior.

For OARs (Figure 2), dose differences increased with a larger shift. The isocenter shift in the R, P and I directions increased the dose to OARs. For Lung L (Figure 2), V_40Gy_ (Figure 2A) in the 2A-VMAT plans showed a stronger robustness. There were no appreciable differences in ∆V_20__Gy_ (Figure 2B), ∆V_5__Gy_ (Figure 2C) and ∆D_mean_ (Figure 2D) for Lung L with both techniques. The 2A-VMAT plans exhibited a slight sensitivity to the L-R shift, while the 7F-H-IMRT plans to the S-I shift. For heart (Figure 2), V_40__Gy_ (Figure 2E) in the 2A-VMAT plans showed an appreciably stronger robustness. Greater dose differences were observed in ∆V_20__Gy_ (Figure 2F), ∆V_5__Gy_ (Figure 2G), and ∆D_mean_ (Figure 2H) for heart in the 7F-H-IMRT plans. Shifts in the S-I directions exerted the greatest effect on the heart.

**Figure 3:**
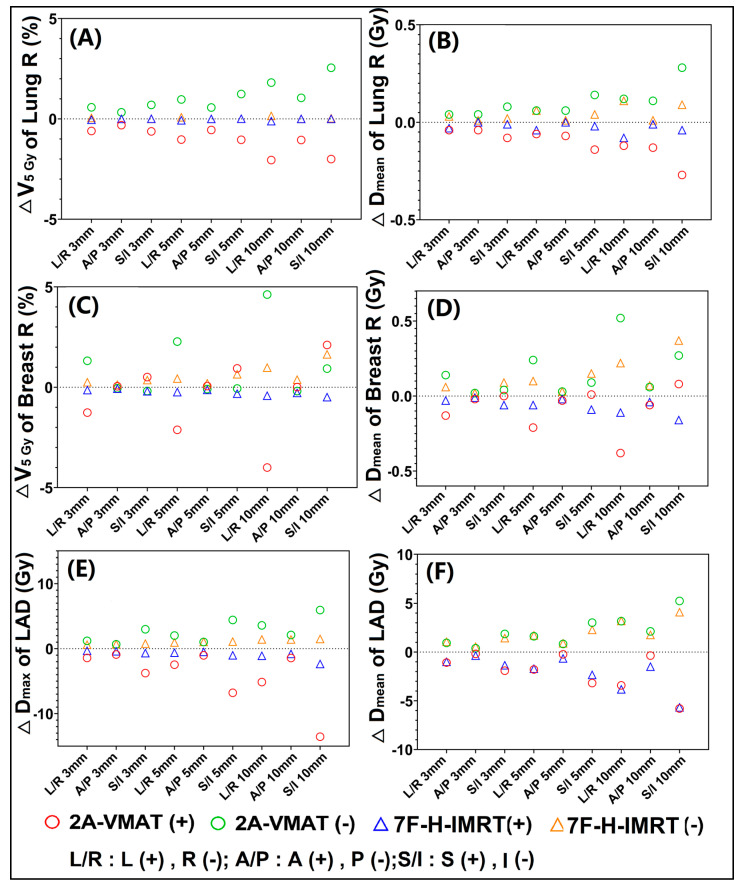
Dose difference between the reference and perturbed 2A-VMAT and 7F-H-IMRT plans for different isocenter shifts. (**A**) ∆V_5 Gy_ of Lung R; (**B**) ∆D_mean_ of Lung R; (**C**) ∆V_5 Gy_ of Brest R; (**D**) ∆D_mean_ of Brest R; (**E**) ∆D_max_ of LAD; (**F**) ∆D_mean_ of LAD. Lung R, right lung; Breast R, right breast; LAD, left anterior descending artery. V_y Gy_, the volume (in percentage) received by y Gy; D_mean_, the mean dose; D_max_, the max dose; 2A-VMAT, 2-arc volumetric arc modulation radiotherapy; 7F-H-IMRT, 7-field hybrid intensity-modulated radiotherapy; L, left; R, right; A, anterior; P, posterior; S, superior; I, inferior.

For Lung R, increased ∆V_5__Gy_ (Figure 3A) and ∆D_mean_ (Figure 3B) were found in the 2A-VMAT plans. ∆V_5Gy_ was sensitive to the L-R direction, while D_mean_ to the S-I direction. Similarly, both ∆V_5Gy_ (Figure 3C) and ∆D_mean_ (Figure 3D) of Breast R exhibited discernible sensitivity to the L-R direction shift in the 2A-VMAT plans. The 2A-VMAT plans showed more appreciable dose differences in D_max_ (Figure 3E) of LAD. However, no obvious difference was found for D_mean_ (Figure 3F) of LAD. The shift in S-I induced maximum dose differences in LAD for both the 2A-VMAT and 7F-H-IMRT plans. 

For 3 mm, TCP did not discernibly differ in both CTV and CTV Boost with a minor ∆TCP (Table 6). A 5 mm shift in the I direction induced in the 2A-VMAT plans increased the ∆TCP (−0.93%) of CTV. For a 10 mm shift, an appreciable ∆TCP of CTV in the 2A-VMAT plans was observed in the R, A, P, S, and I directions, while only 10 mm shift in the A and S directions induced discernible differences in CTV in the 7F-H-IMRT plans. A slight ∆TCP for CTV Boost was seen in CTV Boost for both techniques, except for the 10 mm shift in the R direction of the 2A-VMAT plans. The 7F-H-IMRT plans showed a higher NTCP reduction (∆NTCP) in Lung L (Table 6) with a 3 mm, 5 mm and 10 mm shift in all directions. Other minimal ∆NTCP differences were seen in Lung R and Heart, and could be considered to be negligible (Table 6).

## 4. Discussion

During the last decade, evidence has accumulated showing that adjuvant radiotherapy for left-sided breast cancer (BC) increases the risk of heart and coronary toxicity [22,23,24]. Highly optimized VMAT showed an improved dose distribution, and heart and LAD sparing, comparing to 3D-CRT, with an unexpected large low-dose volume [13,14]. The results from our study demonstrated that VMAT effectively reduced doses to the heart and left lung in left-sided breast cancer patients treated with whole breast irradiation, with a lower value of NTCP. D_max_ (* *p* = 0.002) and D_mean_ (* *p* = 0.002) of LAD significantly decreased in VMAT plans. VMAT produced a larger volume of low-dose regions in the surrounding normal tissue, and induced higher V_5Gy_ and D_mean_ of contralateral lung and breast. These results were in agreement with previously published studies [25]. 

VMAT produces complex intensity modulation through a combination of MLC leaf motion, gantry rotation, and dose-rate change [26]. Smaller and irregular beam apertures, larger tongue-and groove effects, and a greater extent of modulation of machine parameters were required in high-modulation VMAT plans compared to non-modulate plans [27]. Concerns of robustness were elevated with an increased complexity of the VMAT plans. The highly optimized VMAT plans showed sensitivity of dose delivery to subtle deviations, not only in machine parameters, but also in target motions [15,28]. A previous study reported that the accuracy of dose calculation and delivery might been reduced in highly complex plans [29,30,31]. In our study, when perturbations were introduced, higher dose deviations were observed with higher ∆D_98%_, ∆D_95%_ and ∆D_mean_ values of CTV in the 2A-VMAT plans (Table 3). As the shifts increased, slight deviations were seen in 7F-H-IMRT plans (less than 1.0%), while enlarged dose deviations were observed in the 2A-VMAT plans. With a 10 mm shift, the value of ∆D_98%_ in 2A-VMAT plans was over 10 times higher than those in the 7F-H-IMRT plans. Appreciable ∆D_98%_, ∆D_95%_ in the 2A-VMAT plans induced a higher ∆TCP than the 7F-H-IMRT plans. A possible reason for the 7F-H-IMRT plans showing a greater robustness for PTV was that the tangential fields in the 7F-H-IMRT plans had no complex modulation and increased the robustness, accompanied by higher doses to the heart, left lung, and LAD. As to CTV Boost, a noticeably higher ∆D_mean_ was found in the 7F-H-IMRT plans in spite of lower values of ∆D_98%_ and ∆D_95%_. In radiotherapy using a simultaneous integrated boost (SIB), tangential fields could not guarantee that the CTV Boost can acquire an adequate prescription dose with target shifts. As to OARs, shifts in the R, P, and I directions contributed to an increase in the received dose, bringing the treatment field closer to the OARs. The 2A-VMAT plans showed, not only better dose sparing, but also a greater robustness in a high-dose volume (V_40Gy_) than in a low-dose volume (V_20Gy_ and V_5Gy_) of Lung L and Heart. The 7F-H-IMRT plans showed higher ∆NTCP in Lung L (Table 5). The 2A-VMAT plans decreased the max dose of LAD but exhibited a lower robustness. Right lung and right breast showed an apparently higher robustness in the 7F-H-IMRT plans due to the lower dose compared to the 2A-VMAT plans. The ∆NTCP in other OARs could be considered negligible. The shifts in the A-P directions exerted a minimal effect in all OARs. From the above results, it is not difficult to show that the balance between plan modulation and plan robustness in high-degree modulated techniques should be reached. Robustness quantification should be taken into consideration. 

Treatment plan robustness is the degree of resiliency of the required dose distribution to these uncertainties and varies based on the treatment site, technique, and method. Yock’s [18] report reviewed robustness analysis methods and their dosimetric effects, to promote reliable plan evaluation and dose reporting, particularly during clinical trials conducted across different institutions and treatment modalities. This plan-robustness quantification method is recommended for application in clinical treatments. 

Robust optimization was applied to account for position uncertainties relative to the target volume during treatment delivery. Instead of a fixed CTV-to-PTV margin, robust optimization generated scenario-based plans in which geometric uncertainties were taken into consideration [32,33,34]. The best scenario could be obtained by reaching a balance between dose coverage robustness and OAR coverage. 

In addition, a respiration-induced target motion (translation, rotation, and deformation) of several centimeters has been observed in liver, lung, and breast cancer patients [35]. Respiration-induced motion is a significant factor for geometric and dosimetric uncertainties during treatment planning and delivery [36]. Studies should focus on how to reduce the dose delivery inaccuracy caused by these uncertainties. Firstly, motion mitigation techniques should be adopted to decrease the target motion during radiotherapy. Optical surface-guided radiotherapy (SGRT), combining deep inspiration breath-hold (DIBH) technique obtained, not only lower irradiated heart and LAD doses by pushing them away, but also reproducible decreased target motion [37,38]. Jacobson [39] explored an innovative strategy to reduce the tumor motion associated with the DIBH technique using continuous positive airway pressure (CPAP), which is the administration of positive pressure to the airways during the entire respiratory cycle. Therefore, a precision tumor motion management could be a key in the precise radiotherapy. 

Thirdly, we noticed that shifts in the S-I directions exerted the greatest effect for both CTV and CTV Boost. The CTV-to-PTV margin method was adopted based on the Van Herk margin formula [40] in margin-based treatment planning, to ensure the dose coverage of CTV through blurring dose distribution induced by systematic setup errors. This could possibly result in overdosing or underdosing [41,42]. Miao [43] proposed a nonuniform CTV-to-PTV margin method to minimize the volume of PTVs, based on a statistical model considering both the conventional translational error and the additional rotational uncertainty. Gordon [44] proposed a coverage-based treatment planning (CBTP) to produce treatment plans that ensure target coverage by adjusting the margin until the specified CTV coverage is achieved, accompanied by CTV coverage probability analysis. The non-uniform margin method may offer a new direction to improve the dose distribution.

Among this study’s limitations, it is important to highlight that the shifts were adopted in a single direction and calculated 15 times in each perturbed plan to visualize the dose deviation in the one direction. Practically, the respiration-induced relative motion was the combination in several directions in a regular breathing cycle [45]. During the breathing cycle, the tumor moved out of PTV and thus received lower dose in one half of the cycle, then moved back to the central of PTV in the other half of the cycle [46]. Tumor movement around a systematic offset had a similar blurring effect as the set-up errors for the respiration-induced relative motion are not random events. 

## 5. Conclusions

Shifts in the S-I directions exerted the greatest effect for both CTV and CTV Boost. As to OARs, shifts in right, posterior, and inferior directions contributed to an increased dose, due to their proximity to the treatment fields. The dose distribution delivered to the patient depends, not only on the calculated dose distribution, but also on the plan robustness and complexity. Highly modulated VMAT plans exhibited decreased robustness compared to non-modulated techniques. The quantification of plan robustness is recommended in clinical treatment. The robust optimization and motion management may improve the accuracy of dose delivery.

## Figures and Tables

**Table 1:** Clinical and demographic characteristics in our patient cohort.

Patient Number	Age	Patient Anatomy		Stage
		PTV Volume (cm^3^)	PTV Boost Volume (cm^3^)	
1	53	737.3	81.4	T1N0M0
2	59	1011.4	126.8	T1N0M0
3	39	523.2	83	T1N0M0
4	52	1600.9	125.4	T1N0M0
5	57	321.7	59.6	T2N0M0
6	62	524.3	74.5	T1N0M0
7	47	874	156.7	T1N0M0
8	55	503.6	137.8	T1N0M0
9	64	693.3	98.2	T1N0M0
10	48	408.9	55.2	T1N0M0
Mean	54	719.9	99.9	-
Median	54	608.8	90.6	-

Abbreviation: PTV, planning target volume; PTV Boost, planning target volume boost.

**Table 2:** Dosimetric parameters of PTV, PTV Boost, CTV and CTV Boost in 10 patients. The results were explicit by mean and range (minimum–maximum). In bold * *p* values < 0.05 and ** *p* values < 0.01.

	Evaluated Items	2A-VMAT(Gy)	7F-H-IMRT(Gy)	*p*-Value
PTV Boost	D_2cc_	52.61 (51.98–53.30)	52.41 (51.82–52.89)	0.06
	D_98%_	48.96 (48.76–49.15)	49.08 (48.54–49.08)	0.19
	D_mean_	51.21 (50.75–51.93)	51.00 (50.62–51.59)	0.11
CTV Boost	D_98%_	49.74 (49.18–50.08)	49.75 (48.32–50.37)	0.62
	D_95%_	50.62 (49.96–50.18)	50.07 (49.20–50.61)	0.69
	D_mean_	51.54 (40.91–52.37)	51.28 (50.93–51.76)	0.13
	TCP	97.74	97.66	0.11
PTV	D_98%_	42.98 (41.97–46.39)	42.39 (41.70–42.98)	0.43
	D_95%_	43.58 (43.36–44.35)	43.81 (42.83–44.21)	0.19
	D_mean_	46.51 (45.76–48.10)	46.40 (45.77–47.63)	0.23
CTV	D_98%_	43.64 (43.12–44.45)	42.82 (41.53–42.82)	** 0.002
	D_95%_	44.24 (43.83–45.18)	43.81 (42.83–44.21)	** 0.002
	D_mean_	46.96 (46.00–48.58)	46.70 (45.96–47.60)	* 0.05
	TCP	95.44	95.24	0.13

Abbreviation: PTV, planning target volume; PTV Boost, planning target volume boost; CTV, clinical target volume; CTV Boost, clinical target volume boost; TCP, tumor control probability; D_x%_ represented the dose (in Gy) received by x% of the volume, V_y Gy_ the volume (in percentage) received by y Gy, D_2cc_ the dose (in Gy) received by a volume of 2 cm^3^; 2A-VMAT, 2-arc volumetric arc modulation radiotherapy; 7F-H-IMRT, 7-field hybrid intensity-modulated radiotherapy.

**Table 3:** Dosimetric parameters of Lung L, Lung R, Heart, Breast R and LAD in 10 patients. The results were explicit by mean and range (minimum–maximum). In bold * p values < 0.05 and ** *p* values < 0.01.

	Evaluated Items	2A-VMAT(%/Gy)	7F-H-IMRT(%/Gy)	*p*-Value
Lung L	V_40 Gy_	1.49 (0.02–3.36)	7.72 (1.04–11.79)	** 0.002
	V_20 Gy_	13.58 (8.83–20.56)	16.86 (6.64–25.08)	** 0.001
	V_5 Gy_	44.62 (28.15–66.84)	38.45 (31.11–52.74)	* 0.05
	D_mean_	8.72 (6.86–12.27)	9.65 (7.31–12.69)	* 0.04
	NTCP	0.07	0.18	0.08
Lung R	V_5 Gy_	10.96 (0.01–30.62)	0.14 (0.00–0.72)	** 0.002
	D_mean_	2.47 (1.28–4.11)	0.39 (0.09–1.02)	** 0.002
	NTCP	1.97 × 10^−5^	2.08 × 10^−6^	** 0.002
Heart	V_40 Gy_	0.18 (0.00–0.98)	3.76 (1.28–6.24)	** 0.002
	V_20 Gy_	5.15 (1.18–40.90)	9.58 (4.43–17.35)	** 0.002
	V_5 Gy_	22.37 (12.08–31.62)	24.13 (14.22–41.43)	0.43
	D_mean_	4.95 (3.10–6.92)	6.06 (4.06–9.66)	** 0.001
	NTCP	1.89 × 10^−11^	5.63 × 10^−7^	** 0.002
Breast R	V_5 Gy_	17.01 (4.48–33.45)	0.67 (0.00–2.57)	** 0.002
	D_mean_	3.37 (1.51–4.67)	0.63 (0.26–1.01)	** 0.002
LAD	D_max_	40.01 (34.96–44.19)	45.50 (42.89–48.50)	** 0.002
	D_mean_	18.97 (8.33–29.84)	26.34 (12.26–38.62)	** 0.002

Abbreviation: Lung L, left lung; Lung R, right lung; Breast R, right breast; NTCP, normal tissues complication probability; V_y,Gy_ the volume (in percentage) received by y Gy, D_mean_, the mean dose; D_max_, the max dose; 2A-VMAT, 2-arc volumetric arc modulation radiotherapy; 7F-H-IMRT, 7-field hybrid intensity-modulated radiotherapy.

**Table 4:** Mean value and range of clinical target volume (CTV) dose–volume histogram (DVH) dosimetric parameters absolute difference between the reference and perturbed 2A-VMAT and 7F-H-IMRT plans for different isocenter shifts. Data in bold: ∆D > 1 Gy.

Uncertainty	CTV					
	∆D_98%_		∆D_95%_		∆D_mean_	
	2A-VMAT (Gy)	7F-H-IMRT (Gy)	2A-VMAT (Gy)	7F-H-IMRT (Gy)	2A-VMAT (Gy)	7F-H-IMRT (Gy)
L (3 mm)	0.87 (0.18–2.11)	0.12 (0.001–0.26)	0.25 (0.14–0.37)	0.11 (0.00–0.30)	0.20 (0.9–0.31)	0.19 (0.05–0.46)
R (3 mm)	0.43 (0.00–0.90)	0.13 (0.01–0.24)	0.52 (0.28–0.89)	0.15 (0.04–0.27)	0.29 (0.15–0.47)	0.20 (0.01–0.88)
A (3 mm)	0.49 (0.01–1.22)	0.07 (0.01–0.21)	0.03 (0.00–0.09)	0.07 (0.01–0.18)	0.04 (0.00–0.10)	0.09 (0.02–0.26)
P (3 mm)	0.08 (0.00–0.25)	0.09 (0.00–0.31)	0.08 (0.01–0.14)	0.10 (0.00–0.32)	0.04 (0.01–0.10)	0.10 (0.02–0.27)
S (3 mm)	**1.57 (0.12–3.69)**	0.10 (0.02–0.25)	0.29 (0.12–0.40)	0.11 (0.01–0.29)	0.21 (0.08–0.38)	0.18 (0.03–0.48)
I (3 mm)	0.84 (0.07–2.71)	0.12 (0.03–0.30)	0.92 (0.41–2.32)	0.18 (0.02–0.40)	0.40 (0.27–0.53)	0.12 (0.02–0.23)
L (5 mm)	**2.00 (0.13–4.64)**	0.15 (0.02–0.27)	0.31 (0.14–0.57)	0.14 (0.01–0.29)	0.29 (0.15–0.48)	0.26 (0.05–0.60)
R (5 mm)	**1.46 (0.28–3.79)**	0.17 (0.03–0.39)	**1.22 (0.53–2.13)**	0.25 (0.05–0.44)	0.63 (0.28–0.95)	0.46 (0.01–2.60)
A (5 mm)	**1.45 (0.07–4.64)**	0.05 (0.01–0.12)	0.09 (0.00–0.25)	0.07 (0.00–0.13)	0.08 (0.00–0.20)	0.11 (0.03–0.24)
P (5 mm)	0.50 (0.03–1.22)	0.10 (0.00–0.32)	0.20 (0.06–0.38)	0.11 (0.00–0.33)	0.07 (0.01–0.18)	0.11 (0.00–0.28)
S (5 mm)	**3.37 (0.10–7.94)**	0.21 (0.00–0.63)	0.29 (0.12–0.51)	0.15 (0.03–0.37)	0.30 (0.04–0.57)	0.24 (0.04–0.62)
I (5 mm)	**2.77 (0.81–6.17)**	0.17 (0.03–0.52)	**2.45 (0.97–5.33)**	0.34 (0.02–0.67)	0.91 (0.53–1.35)	0.22 (0.02–0.50)
L (10 mm)	**5.97 (40.52–43.57)**	0.60 (0.02–1.21)	0.45 (0.06–1.29)	0.33 (0.01–0.80)	0.87 (0.07–4.71)	0.39 (0.00–0.86)
R (10 mm)	**7.36 (31.74–39.51)**	0.45 (0.07–0.83)	**5.70 (2.13–8.84)**	0.63 (0.03–1.01)	**1.93 (0.88–3.59)**	**1.02 (0.05–5.04)**
A (10 mm)	**6.40 (40.64–43.42)**	0.38 (0.03–0.83)	0.44 (0.14–1.00)	0.29 (0.07–0.50)	0.24 (0.04–0.54)	0.14 (0.03–0.25)
P (10 mm)	**3.90 (40.55–43.43)**	0.14 (0.00–0.29)	0.94 (0.46–1.61)	0.15 (0.07–0.25)	0.30 (0.06–0.55)	0.24 (0.03–0.98)
S (10 mm)	**10.28 (33.43–42.70)**	**1.80 (0.11–4.37)**	**1.89 (0.30–6.10)**	0.82 (0.01–2.30)	0.82 (0.19–2.02)	0.51 (0.02–0.89)
I (10 mm)	**10.21 (27.46–35.46)**	0.64 (0.17–1.22)	**9.14 (4.97–14.22)**	0.89 (0.12–1.29)	**2.99 (1.63–5.91)**	0.60 (0.12–1.96)

CTV, clinical target volume; D_x%_ represented the dose (in Gy) received by x% of the volume D_mean_ represented the mean dose (in Gy). 2A-VMAT, 2-arc volumetric arc modulation radiotherapy; 7F-H-IMRT, 7-field hybrid intensity-modulated radiotherapy. ∆D represented the absolute dose difference which was calculated by the absolute value of the minimum value subtracted from the original value. L, left; R, right; A, anterior; P, posterior; S, superior; I, inferior.

**Table 5:** Mean value and range of clinical target volume boost (CTV Boost) dose–volume histogram (DVH) of the dosimetric parameters’ absolute difference between the reference and perturbed 2A-VMAT and 7F-H-IMRT plans for different isocenter shifts. Data in bold: ∆D_x%_ > 1 Gy.

Uncertainty	CTV Boost					
	∆D_98%_		∆D_95%_		∆D_mean_	
	2A-VMAT (Gy)	7F-H-IMRT (Gy)	2A-VMAT (Gy)	7F-H-IMRT (Gy)	2A-VMAT (Gy)	7F-H-IMRT (Gy)
L (3 mm)	0.83 (0.13–2.11)	0.31 (0.06–0.61)	0.36 (0.15–0.71)	0.27 (0.00–0.64)	0.22 (0.02–0.48)	0.27 (0.00–0.56)
R (3 mm)	0.42 (0.00–1.31)	0.25 (0.02–0.63)	0.42 (0.15–1.13)	0.23 (0.03–0.60)	0.27 (0.10–0.47)	0.99 (0.07–7.15)
A (3 mm)	0.38 (0.03–1.02)	0.13 (0.01–0.25)	0.07 (0.01–0.12)	0.11 (0.02–0.34)	0.07 (0.00–0.15)	0.13 (0.01–0.47)
P (3 mm)	0.06 (0.00–0.31)	0.18 (0.01–0.59)	0.11 (0.00–0.35)	0.13 (0.00–0.40)	0.06 (0.01–0.13)	0.25 (0.00–1.23)
S (3 mm)	**1.33 (0.03–3.69)**	0.31 (0.11–0.61)	0.37 (0.02–0.98)	0.28 (0.05–0.53)	0.22 (0.05–0.35)	0.36 (0.03–1.34)
I (3 mm)	0.41 (0.07–0.78)	0.37 (0.03–0.65)	0.58 (0.29–1.68)	0.32 (0.02–0.55)	0.34 (0.05–0.74)	0.40 (0.02–1.83)
L (5 mm)	**1.67 (0.16–4.24)**	0.58 (0.02–1.26)	0.57 (0.12–1.69)	0.45 (0.05–1.04)	0.32 (0.18–0.82)	0.42 (0.01–0.74)
R (5 mm)	**1.36 (0.28–4.16)**	0.40 (0.06–1.18)	**1.09 (0.41–3.42)**	0.41 (0.17–0.97)	0.82 (0.16–3.13)	**2.65 (0.08–18.15)**
A (5 mm)	0.97 (0.03–2.60)	0.24 (0.05–0.50)	0.11 (0.03–0.21)	0.14 (0.05–0.31)	0.10 (0.02–0.21)	0.15 (0.01–0.51)
P (5 mm)	0.35 (0.03–0.86)	0.22 (0.07–0.63)	0.24 (0.02–0.63)	0.15 (0.00–0.33)	0.17 (0.01–0.47)	0.57 (0.02–3.71)
S (5 mm)	**2.78 (0.10–7.94)**	0.53 (0.15–1.15)	0.62 (0.03–2.55)	0.43 (0.12–0.71)	0.36 (0.05–0.63)	0.81 (0.05–3.01)
I (5 mm)	**1.57 (0.81–3.23)**	**0.69 (0.24–1.14)**	**1.38 (0.75–3.85)**	0.61 (0.08–1.02)	0.74 (0.23–1.47)	0.77 (0.10–3.67)
L (10 mm)	**5.55 (0.34–11.97)**	**1.73 (0.61–3.21)**	**1.68 (0.17–5.48)**	**1.16 (0.06–2.35)**	**1.94 (0.04–14.86)**	0.72 (0.02–1.42)
R (10 mm)	**6.67 (1.40–12.45)**	**1.64 (0.40–3.88)**	**4.53 (1.08–9.24)**	**1.36 (0.33–2.75)**	**2.31 (0.38–9.16)**	**5.01 (0.51–28.10)**
A (10 mm)	**5.21 (0.38–12.28)**	**2.29 (0.84–4.01)**	**1.09 (0.27–2.62)**	**1.47 (0.22–2.73)**	0.32 (0.03–0.84)	0.29 (0.01–0.65)
P (10 mm)	**3.41 (0.64–7.52)**	**1.26 (0.01–2.84)**	**1.54 (0.35–2.84)**	0.75 (0.06–2.16)	0.89 (0.11–2.75)	**1.39 (0.16–8.49)**
S (10 mm)	**8.13 (0.57–20.81)**	**2.06 (0.63–4.29)**	**1.98 (0.03–8.15)**	**1.25 (0.01–2.68)**	**1.15 (0.08–5.94)**	**2.59 (0.22–7.84)**
I (10 mm)	**7.18 (2.61–11.84)**	**2.22 (0.76–4.65)**	**5.24 (2.08–11.47)**	**1.84 (0.56–3.70)**	**2.44 (0.1.47–4.35)**	**1.35 (0.00–5.14)**

CTV Boost, clinical target volume boost; D_x%_ represented the dose (in Gy) received by x% of the volume, D_mean_ represented the mean dose (in Gy).; 2A-VMAT, 2-arc volumetric arc modulation radiotherapy; 7F-H-IMRT, 7-field hybrid intensity-modulated radiotherapy. ∆D_x%_ represented the absolute dose difference which was calculated by the absolute value of the minimum value subtracted from the original value. L, left; R, right; A, anterior; P, posterior; S, superior; I, inferior.

**Table 6:** Tumor control probability reduction (∆TCP) of CTV and CTV Boost, normal tissue complication probability reduction (∆NTCP) of OARs.

Shift	∆TCP(%)				∆NTCP(%)					
	CTV		CTV Boost		Lung L		Lung R		Heart	
	2A-VMAT	7F-H-IMRT	2A-VMAT	7F-H-IMRT	2A-VMAT	7F-H-IMRT	2A-VMAT	7F-H-IMRT	2A-VMAT	7F-H-IMRT
L (3 mm)	0.10	0.09	0.03	0.02	0.03	0.06	3.77 × 10^−6^	1.14 × 10^−7^	9.82 × 10^−12^	3.15 × 10^−8^
R (3 mm)	−0.16	0.02	−0.06	0.07	0.06	0.12	4.08 × 10^−6^	1.48 × 10^−7^	3.55 × 10^−11^	1.15 × 10^−7^
A (3 mm)	−0.01	0.04	0.01	0.03	0.01	0.02	2.11 × 10^−6^	1.05 × 10^−8^	3.79 × 10^−12^	1.97 × 10^−8^
P (3 mm)	−0.02	0.07	−0.01	0.04	0.01	0.04	2.14 × 10^−6^	2.90 × 10^−8^	7.93 × 10^−12^	5.67 × 10^−8^
S (3 mm)	0.11	0.14	0.05	0.09	0.03	0.07	5.64 × 10^−6^	6.60 × 10^−8^	1.14 × 10^−11^	3.59 × 10^−8^
I (3 mm)	−0.30	−0.03	−0.07	−0.02	0.05	0.15	5.95 × 10^−6^	9.55 × 10^−8^	7.30 × 10^−11^	1.73 × 10^−7^
L (5 mm)	0.14	0.12	0.02	0.02	0.05	0.09	6.33 × 10^−6^	1.88 × 10^−7^	1.20 × 10^−11^	4.13 × 10^−8^
R (5 mm)	−0.39	0.00	−0.16	0.11	0.13	0.23	6.90 × 10^−6^	2.48 × 10^−7^	1.01 × 10^−10^	2.79 × 10^−7^
A (5 mm)	−0.05	0.03	0.01	0.02	0.01	0.04	3.74 × 10^−6^	1.79 × 10^−8^	5.12 × 10^−12^	2.99 × 10^−8^
P (5 mm)	−0.06	0.07	−0.02	0.04	0.02	0.06	3.71 × 10^−6^	4.31 × 10^−8^	1.75 × 10^−11^	1.17 × 10^−7^
S (5 mm)	0.12	0.18	0.07	0.12	0.04	0.11	9.43 × 10^−6^	1.10 × 10^−7^	1.29 × 10^−11^	4.43 × 10^−8^
I (5 mm)	−0.93	−0.10	−0.14	−0.05	0.11	0.30	1.03 × 10^−5^	1.59 × 10^−7^	2.90 × 10^−10^	4.97 × 10^−7^
L (10 mm)	0.05	0.01	−0.16	−0.03	0.06	0.13	1.20 × 10^−5^	3.40 × 10^−7^	1.59 × 10^−11^	4.84 × 10^−8^
R (10 mm)	−2.65	−0.11	−1.03	0.08	0.49	0.67	1.35 × 10^−5^	5.27 × 10^−7^	9.13 × 10^−10^	1.64 × 10^−6^
A (10 mm)	−9.43	−3.26	−0.08	−0.09	0.02	0.07	7.65 × 10^−6^	3.40 × 10^−8^	6.60 × 10^−12^	4.28 × 10^−8^
P (10 mm)	−2.88	0.04	−0.19	−0.02	0.05	0.13	7.16 × 10^−6^	8.31 × 10^−8^	7.94 × 10^−11^	4.84 × 10^−7^
S (10 mm)	−1.32	−3.65	0.02	0.28	0.06	0.15	1.77 × 10^−5^	2.01 × 10^−7^	1.36 × 10^−11^	4.90 × 10^−8^
I (10 mm)	−9.16	−0.31	−0.58	−0.19	0.36	0.98	2.07 × 10^−5^	3.43 × 10^−7^	7.44 × 10^−9^	4.53 × 10^−6^

CTV Boost, clinical target volume boost; 2A-VMAT, 2-arc volumetric arc modulation radiotherapy; 7F-H-IMRT, 7-field hybrid intensity-modulated radiotherapy. ∆Dx represented the absolute dose difference which was calculated by the absolute value of the minimum value subtracted from the original value. L, left; R, right; A, anterior; P, posterior; S, superior; I, inferior.

## Data Availability

The datasets used and/or analyzed during the current study are available from the corresponding author upon reasonable request.
